# Aesthetic chills mitigate maladaptive cognition in depression

**DOI:** 10.1186/s12888-023-05476-3

**Published:** 2024-01-10

**Authors:** Felix Schoeller, Abhinandan Jain, Vladimir Adrien, Pattie Maes, Nicco Reggente

**Affiliations:** 1 Massachusetts Institute of Technology, Cambridge, MA USA; 2 Institute for Advanced Consciousness Studies, Santa Monica, CA USA; 3 Department of Infectious and Tropical Diseases, AP-HP, Avicenne Hospital, Université Sorbonne Paris Nord, Bobigny, F-93000 France; 4 Institute of Psychiatry and Neuroscience of Paris (IPNP), Université Paris Cité, Inserm UMR-S 1266, Team Membrane Traffic in Healthy & Diseased Brain, Paris, 75014 France

**Keywords:** Depression, Schema, Shame, Psychedelics, Emotional breakthrough, Emotion, Chills

## Abstract

**Background:**

Depression is a major global health challenge, affecting over 300 million people worldwide. Current pharmacological and psychotherapeutic interventions have limited efficacy, underscoring the need for novel approaches. Emerging evidence suggests that peak emotional experiences characterized by awe, transcendence, and meaning hold promise for rapidly shifting maladaptive cognitive patterns in depression. Aesthetic chills, a peak positive emotion characterized by physical sensations such as shivers and goosebumps, may influence reward-related neural pathways and hold promise for modifying core maladaptive beliefs rooted in early adverse experiences.

**Methods:**

We enrolled 96 patients diagnosed with major depressive disorder. A validated database of multimedia known to elicit chills responses (ChillsDB) was used for stimulus presentation. Participants’ emotional responses were assessed using the Emotional Breakthrough Inventory (EBI), while shifts in self-schema were measured via the Young Positive Schema Questionnaire (YSPQ).

**Results:**

The study found that chill-inducing stimuli have the potential to positively influence the core schema of individuals with depression, impacting areas of self-related beliefs. The associated phenomenology triggered by chills appears to share similarities with the altered states of consciousness induced by psychedelic substances like psilocybin.

**Conclusions:**

These preliminary results suggest that the biological processes involved in aesthetic chills could be harnessed as a non-pharmacological intervention for depression. However, further investigation is necessary to comprehensively understand the neurophysiological responses to chills and to evaluate the practicality, effectiveness, and safety of utilizing aesthetic chills as a preventive measure in mental health care.

## Introduction

Depression is a debilitating mental illness characterized by emotional numbness and a generalized lack of motivation or pleasure in life [1, 2]. Depressed patients often exhibit patterns of constant negative rumination about themselves and others, as well as social isolation [1–3]. Depression is associated with dysregulation in the brain’s mesocortical and mesolimbic reward pathways, particularly in areas like the ventral tegmental area (VTA) and the nucleus accumbens (NAc) [4, 5]. These areas, rich in dopaminergic neurons, are crucial for experiencing pleasure and motivation [4, 6]. Such neural changes can diminish a patient’s ability to experience pleasure and contribute to core aspects of depressive symptomatology, such as feelings of worthlessness, hopelessness, and an inability to envision a positive future [6, 7]. Psychotherapy for these patient populations typically attempts to stop the self-reinforcing cycle of negative emotions by addressing the patient’s core maladaptive beliefs, a process known as cognitive restructuring [8–10]. Specifically, evidence shows that schema related to self-image play an essential role in depression and should be the main focus of intervention [9, 11]. In recent years, there has been considerable interest in psychedelic compounds (e.g., psilocybin, lysergic acid diethylamide [LSD]) and their extraordinary phenomenology as a means to induce flexibility in such core beliefs [12], sometimes leading to considerable transformation and behavioral change [13, 14]. Evidence indicates that psychedelics alleviate the symptoms of depression, including in long-term treatment-resistant patients [15–17]. Crucially, subjective experience has been found to be a major predictor of long-term therapeutic outcomes and patient well-being [18, 19]. However, these peak experiences typically require years of psychotherapeutic support and are difficult to reproduce in the absence of psychotomimetic drugs, which may carry contraindications and potential side effects.

Here, we investigated whether the peak experience of aesthetic chills could mimic some of these effects and thereby induce changes in deep-seated models of the self to mitigate shame (i.e., foster self-acceptance) in patients with a depressed mood. Aesthetic chills (thereafter “chills”) are a peak emotional response induced by powerful, sometimes life-changing stimuli [20–23]. They are characterized by a specific bodily response of thermoregulatory mechanisms such as shivers and goosebumps [21, 24–26] and discrete neural correlates associated with reward and well-being [21, 24–27]. Stimuli known to induce chills include music [28], film [29], speech [30], as well as secular and religious rituals [20]. Notably, preliminary evidence suggests that musical chills may induce psychological insight [31], a core phenomenological component of psychedelic experiences and psychotherapeutic work [19, 32]. As a strong hedonic response to emotional stimuli engaging the brain reward system [21, 27], chills may hold scientific and clinical potential for reward-related or dopaminergic illnesses such as depression [33, 34]. However, not much is known about chills and psychopathology [35]. The goal of this study is to examine the effects of chills stimulation on maladaptive cognitions in subjects clinically diagnosed with depression. We investigated whether (1) chill-inducing content may affect the core schema of depressed patients (specifically shame and self-acceptance) and (2) the mechanism at play during the chills response may resemble the form of experience induced by the psychedelic and psychotherapeutic experience, leading to similar positive outcomes for affective disorders patients. To measure the psychedelic phenomenology we used the recently developed Emotional Breakthrough Inventory which typically predicts long-term therapeutic outcomes in psychedelic-assisted psychotherapy [18], and to assess positive belief change after the exposure we used Young Positive Schema Questionnaire (YSPQ) [36].

To test the effects of chills videos on depression and self-schema, we used two stimuli from ChillsDB, a recently constituted chills stimuli database [22]: one motivational video specifically targeting self-schema through emotional invectives to the listener and a viral commercial video targeting pro-social feelings and known to elicit chills and tears. Both have been shown in past studies to elicit chills in 80% of subjects as well as powerful emotional reactions [37]. To estimate the subjective effects of chills and assess to what extent they may resemble psychedelic or psychotherapeutic experiences, we used the recently constituted Emotional Breakthrough Inventory (EBI; [18]). To assess positive belief change after exposure, we used the Young Positive Schema Questionnaire (YSPQ; [36]). We hypothesized that (a) chill-inducing stimuli will increase self-acceptance and decrease shame in depressed individuals, as measured by the self-acceptance schema on the YSPQ and (b) the experience of chills will correlate with greater emotional breakthrough on the EBI. Testing these hypotheses will provide initial evidence on the effects of aesthetic chills on maladaptive cognition in depression, and determine if the chills mechanisms can potentially mimic psychedelic-assisted psychotherapy to modify entrenched negative self-schemas.

## Methods

### Participants

This study recruited a cohort of 96 participants (52 females; mean age = 37 years, SD = 14) through an online platform with comprehensive pre-screening features commonly used to recruit participants (Prolific). Prolific is a specialized online platform designed to connect researchers with a diverse global pool of participants for research studies, offering tailored participant recruitment through a range of pre-screening tools including mental health diagnoses, medication, age, gender identity, nationality, and employment status [38]. All participants reported a prior physician-diagnosed depression. Among them, 90.7% were currently undergoing therapy (N = 78), and 97.7% were on medication (selective serotonin reuptake inhibitor), N = 84. To ensure sample homogeneity and confirm that all participants were experiencing depression at the time of the intervention, we excluded 10 individuals who reported that they were neither undergoing treatment nor taking medication currently. Additionally, to ensure clarity in communication and reliability of responses, participants were screened for native-level English proficiency.

### Procedure

After being informed about the experiment and signing the consent form, participants were randomly assigned to one of the two experimental conditions (Stimulus 1 vs. Stimulus 2). Participants were first asked to answer questions about demographics (age, gender, nationality, ethnicity), and questions from 5 YSPQ schemas: Self-acceptance, Trust, Social Belonging, Self-reliance/competence, and Emotional Fulfillment. Following the circumplex model of emotion [39], participants were asked to report their current mood in feeling “Extremely Unpleasant” to “Extremely Pleasant” for the valence rating, “Extremely Calm” to “Extremely Excited” for the arousal rating before exposure to the stimulus on a 10 point Likert scale. Immediately after watching the entirety of the stimulus, the participants were asked about their emotional valence and arousal, and some phenomenological questions about the experience (see the self-report subsection). Participants were asked whether they experienced chills, as well as the intensity, frequency, and duration of chills, whether the video reminded them of any personal experience, and to describe the content of the video that generated chills. Participants then answered the EBI and took the same 5 schemas from the YPSQ in a randomized order. Participants were provided with a contact for any further information. The experiment lasted about 15 min.

### Materials

#### Stimuli

**Fig. 1:**
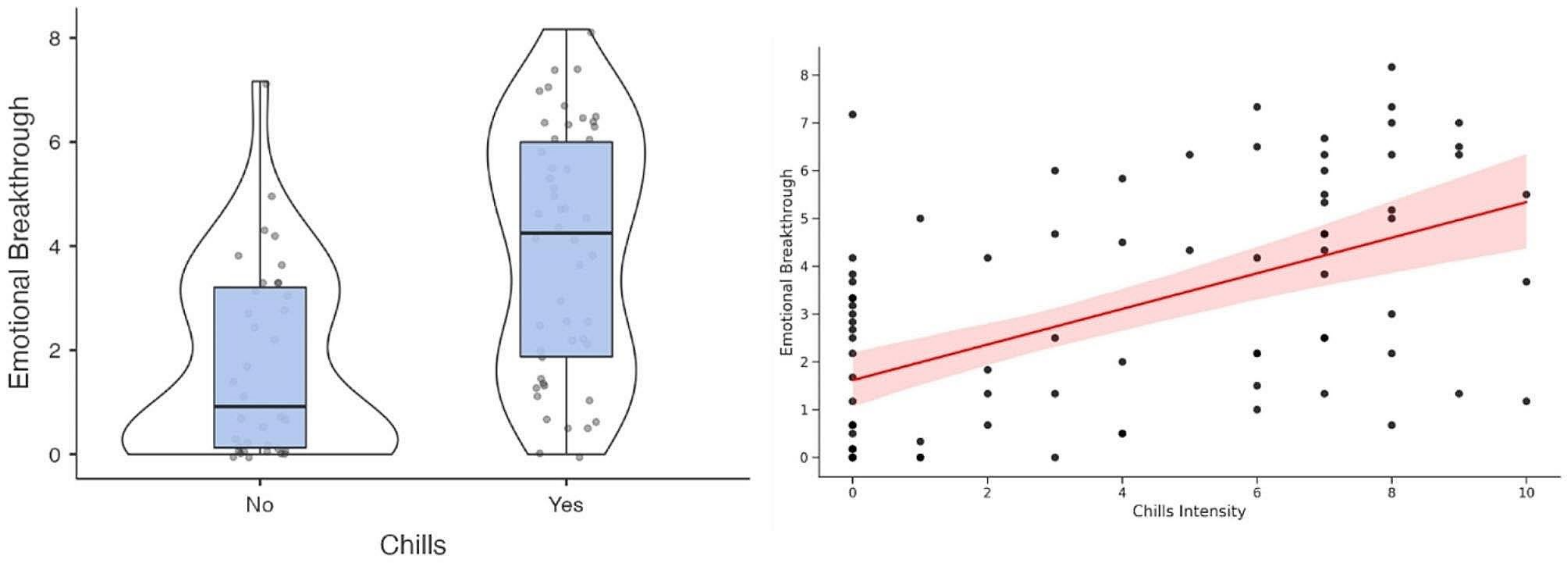
On the left, differences in emotional breakthrough across the chills and non chills conditions. On the right, chills intensity positively correlated with the emotional breakthrough

To identify the stimuli, we used ChillsDB, an open-source database of validated audiovisual stimuli that are known to elicit aesthetic chills (goosebumps, psychogenic shivers) in a US population [22]. The database consists of 204 chills-eliciting videos in three categories: music, film, and speech (see Fig. 1), which were validated across 600 + participants. ChillsDB was built using an ecologically valid method for harnessing chills stimuli “in the wild” by searching for mentions of somatic markers in user comments using algorithms to parse social media platforms (YouTube and Reddit). Two stimuli were extracted from the top six validated videos. Both stimuli have a probability ≥ 0.8 of eliciting chills in a US population. Stimulus 1: “Giving” (Thailand, 2013, 3 min) is a three-minute Thai TV commercial by the TrueMove mobile company. Stimulus 2: The Dream stimulus (6 min 19 s) was selected specifically for its ability to affect the self-image of subjects. It is a medley of motivational speeches by speakers such as Les Brown, Eric Thomas, and Will Smith, accompanied by emotional music.

#### Measures

- Emotional Breakthrough Inventory (EBI)

The EBI was developed using an Internet survey of those who reported using a psychedelic [18]. It is a reliable and validated scale that is positively associated with increases in well-being after a psychedelic experience [18]. The EBI consists of eight statements such as “I felt able to explore challenging emotions and memories” and asks about “emotional release”, “closure”, “emotional breakthrough” and “resolution of conflict”. Participants rated the extent to which they agreed with each statement on a 0–10 scale (with 0 being “No, not more than usual” and 10 being “Yes, entirely or completely”).

b. Young Positive Schema Questionnaire (YPSQ)

The Young Positive Schema Questionnaire (YPSQ; [36]) is a 56-item self-report measure designed to assess 14 positive schema factors: Emotional Deprivation, Abandonment, Mistrust/Abuse, Social Isolation, Defectiveness, Failure, Dependence, Vulnerability to Harm, Enmeshment, Subjugation, Self-Sacrifice, Emotional Inhibition, Unrelenting Standards, and Entitlement. The YPSQ was developed to complement the Young Schema Questionnaire 3 Short Form (YSQ-S3; [10]) which measures early maladaptive or negative schemas. Participants respond to YPSQ items on a 6-point Likert scale ranging from 1 (completely untrue of me) to 6 (describes me perfectly). Scale scores are calculated by summing the items for each subscale. Higher scores indicate greater endorsement of the positive schema. For this study, we measured the schema most often associated with depression [8, 9, 11]. These are Self-acceptance/lovability schema (equivalent to the Shame/effectiveness schema in the original early maladaptive schema (EMS)), the Trust schema (equivalent to Mistrust/abuse in EMS), Social Belonging (Social Isolation and Belonging), Self-reliance/competence (Dependence / Incompetence), Emotional Fulfillment (Emotional Deprivation).

c. Chills Self-Report

Chills were self-reported by the participants through a series of questions regarding their emotional and physiological responses to the stimulus. They responded to binary (Yes/No) questions such as “Did you experience chills?” and “Did you experience goosebumps?“, as well as questions rated on a 0–10 Likert scale, including frequency and intensity. Additionally, a qualitative component involved open-ended responses, asking participants to describe their experience during the video, their description of what caused the chills in the video, and whether the video reminded them of a personal experience, providing a deeper insight into their emotional engagement with the content.

### Ethics

The experiment complied with the Helsinki Declaration and was approved by the Committee on the Use of Humans as Experimental Subjects at the Massachusetts Institute of Technology (MIT). All participants gave their voluntary informed consent and we followed the Ethics Code of the American Psychological Association. All participants were informed about the purpose of the research, their right to decline to participate and to withdraw from the experiment, and the limits of confidentiality. We also provided them with a contact for any questions concerning the research and with the opportunity to ask any questions regarding the phenomenon under study (aesthetic chills) and receive appropriate answers. All participants reacted positively to the experiment and were thankful for the opportunity to learn about the phenomenon.

### Data analysis

We employed descriptive statistics to calculate means and standard deviations for variables like emotional breakthrough and arousal ratings [40]. Following assumption checks, we used the Mann-Whitney U test for inferential analysis, suitable for non-normally distributed data [41], to compare the experiences of participants with and without chills. Additionally, we applied Spearman’s rank correlation coefficient to assess the relationship between chill intensity and emotional breakthrough, a method effective for non-parametric data [42]. This combination of descriptive and inferential statistical approaches aimed to provide a robust analysis of the potential impact of aesthetic chills on depression symptoms. Following the standard reviewer disclosure request endorsed by the Center for Open Science [43], we confirm to have reported all measures, conditions, data exclusions, and how we determined our sample sizes.

## Results

In total, 50 participants reported to have experienced chills (31 in the Giving stimulus, 19 in the Dream stimulus). The Shapiro-Wilk tests indicated non-normality for all variables of interests: Chills Intensity (W = 0.846, *p* < 0.001), Emotional Breakthrough (W = 0.925, *p* < 0.001), Valence Drift (W = 0.943, *p* < 0.001), and Arousal Drift (W = 0.968, *p* = 0.030), indicating the need to use non-parametric tests for subsequent analyses. We found that participants who experienced chills reported a more important emotional breakthrough (M_EBI_=3.91, SD = 2.35) than those who did not (M_EBI_=1.73, SD = 1.85). A Mann Whitney U revealed that this difference is statistically significant (U = 416, *p* < 0.001). The reported intensity of the chills positively correlated with the emotional breakthrough (r(94) = 0.542, *p* < 0.001).

We then proceeded to test for differences in valence and arousal in subjects who experienced chills compared to those who did not. We did not find any significant difference (U = 793, *p* = 0.345) of pre-exposure arousal ratings between subsequently those who reported chills (M_Arousal pre_=3.92, SD = 1.99) and those who did not (M_Arousal pre_=3.44, SD = 1.73). Similarly, we did not find any significant difference (U = 852, *p* = 0.674) of pre-exposure -valence ratings between those who experienced chills (M_Valence pre_=5.88, SD = 1.99) and those who did not (M_Valence pre_=5.69, SD = 1.72), indicating independence between pre exposure emotional state on propensity of getting chills.

**Fig. 2:**
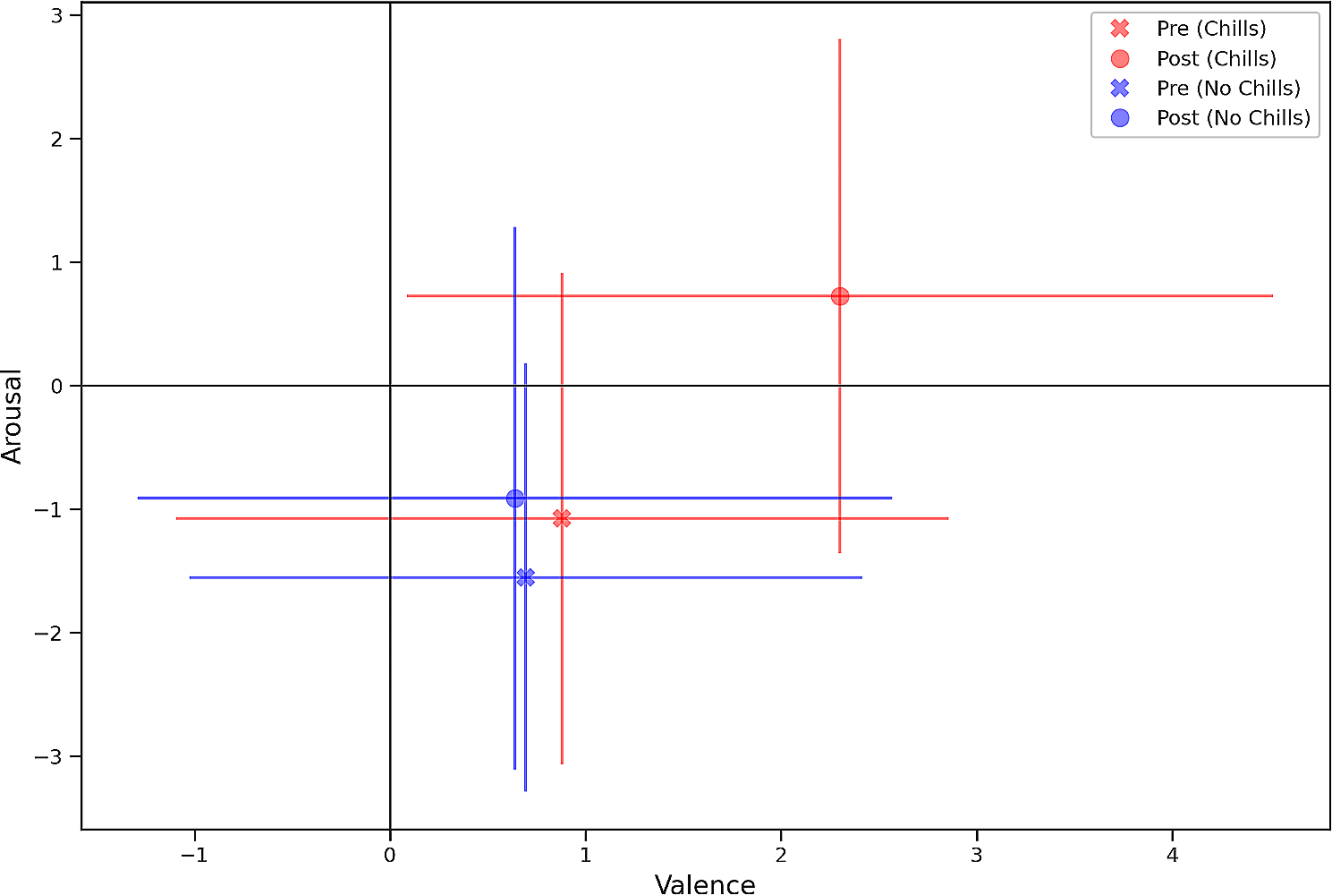
Emotional drift in valence and arousal in chills and no chills conditions. The chills participants reported a greater drift in emotional valence and arousal, and a change from a bottom quadrant to a top quadrant, whereas the participants who did not experience chills remained in the same emotional space

Participants who reported chills also reported greater valence (M_Valence_=7.30, SD = 2.22) and arousal ratings (M_Arousal_=5.72; SD = 2.08) after the stimulus than those who did not (M_Valence_=5.64, SD = 1.93; M_Arousal_=4.08; SD = 2.20) (Fig. 2). Both differences were significant across chills exposure conditions for both valence (U = 493, *p* < 0.001) and arousal ratings (U = 534, *p* = 0.001). We found that an increase in chills intensity correlates with an increase in valence (Spearman’s rho 0.543, < 0.001) and arousal (Spearman’s rho 0.384, < 0.001). Mediation analysis revealed that the intensity of chills significantly predicted the change in valence (total effect = 0.239, SE = 0.0622, Z = 3.84, *p* < 0.001), mediated by emotional breakthrough (indirect effect = 0.122, SE = 0.0430, Z = 2.84, *p* = 0.005).

Finally, upon examining the impact of chills on YPSQ, we applied a Bonferroni correction to account for multiple comparisons across 5 tests, setting the significance threshold at *p* = 0.01. In this analysis, participants who experienced chills demonstrated a statistically significant improvement in self-acceptance (U = 570, *p* = 0.003). Notably, this significant result was not observed in the other five tests, speaking to the specific impact of chills on self-acceptance.

## Discussion and conclusion

In this exploratory study, we tested the effect of two chills stimulations on depressed subjects. We found that the subjects who reported chills displayed significantly greater self-acceptance (i.e., less shame), as measured by YPSQ, and compared to the subjects who did not experience chills. Furthermore, we found that participants who reported chills reported a greater emotional breakthrough, a measure commonly used in psychedelic research to assess the patient’s propensity to experience difficult emotions during the session. Similar to prior studies [37], we also found that chills were significantly correlated with a change in valence and arousal, a positive outcome for depressed patients who ordinarily struggle with anhedonia and lower reward sensitivity [44, 45]. Interestingly, the change in valence score was mediated by the level of emotional breakthrough.

These results suggest that chill-inducing stimuli may have the potential to affect the core schema of depressed patients, specifically in terms of shame and self-acceptance. Core maladaptive beliefs can typically be traced back to adverse childhood experiences (e.g., rape, neglect, abuse) and are often met with considerable resistance by the patient due to the installment of a deep sense of shame and self-deprecation, where the patient perceives himself as inherently defective [46, 47]. The main challenge of psychotherapy is to address these dysfunctional patterns of thoughts and feelings learned early in childhood during states of heightened brain plasticity [48]. Aesthetic chills may potentiate cognitive restructuring through the release of dopamine, and a temporary enhancement of synaptic plasticity, critical for learning and cognitive change [49]. Dopamine’s role in reward prediction error processing is essential for adapting and relearning emotional responses [50], particularly in the context of positive experiences [51] with increased dopamine levels associated with positive affect and enhanced cognitive flexibility [52]. The potent dopaminergic response elicited by aesthetic chills could interrupt negative thought patterns, providing a unique opportunity for positive cognitive and emotional relearning [34, 53, 54], especially with regards to orienting to the semantic content of the stimulus.

Interestingly, we did not find any difference in arousal scores beforehand for participants who reported chills compared to those who did not, contrary to the results of [55], where the statistical difference in arousal score before exposure can be used to predict for the subsequent occurrence of chills in healthy participants. Perhaps, the medication is damping the response.

The mechanism of action during the chills response is not well understood, but our results suggest that it may resemble the form of problem resolution induced by the psychedelic and psychotherapeutic experience [13]. This finding is intriguing, as it suggests that aesthetic chills may be able to mimic some of the therapeutic effects of psychedelics without the use of psychoactive drugs. This may be particularly useful for individuals who are unable or unwilling to use psychedelics for various reasons, such as legal restrictions, safety concerns, or personal preferences.

However, our study has several limitations. First, as an exploratory study, our sample size is limited and may not be representative of the general population. Second, this study lacked formal diagnostic assessment by a clinician at intervention time. Hence, a follow-up study should include detailed clinician interviews (e.g., using the MINI International Neuropsychiatric Interview) for a more robust validation of these preliminary findings. Third, we used a self-report measure to assess the effects of chills on shame and self-acceptance, which may be subject to some bias. Future research should aim to address these limitations and provide more robust evidence for the effects of chills on depression and other mental health conditions. This could be done through larger, more diverse samples, more objective measures of emotional response (e.g., neurophysiological), and more controlled experimental designs. Additionally, research should explore the underlying biological mechanisms of the chills response in the context of depression and its potential therapeutic applications, as well as the potential risks and drawbacks of using aesthetic chills as a therapeutic intervention.

## Conclusion

Aesthetic chills may be a promising avenue for future therapeutic interventions and offer a non-pharmacological and easily accessible means to induce psychoplastogenic states. This preliminary study suggests that chill-inducing stimuli may have the potential to affect the core schema of depressed patients, specifically in terms of shame and self-acceptance. The mechanism of action during the chills response may resemble the form of insight induced by the psychedelic and psychotherapeutic experience, leading to similar positive outcomes for the subject. However, further research is needed to fully understand the immediate and long-term effects of chills on depression and other reward-related or dopaminergic illnesses.

## Data Availability

The data for this study is available on the FigShare repository: Schoeller, F., Jain, A., Adrien, V., Maes, P., & Reggente, N. (2023). Aesthetic Chills Mitigate Maladaptive Cognition In Depression (Version 1). figshare. 10.6084/m9.figshare.24815574.v1.
